# Biochemical and structural response in patients with tall cell papillary thyroid cancer: a dual centre retrospective study: Early Recurrence in Tall Cell PTC

**DOI:** 10.1007/s00405-025-09426-5

**Published:** 2025-05-05

**Authors:** Louis Britten-Jones, Spinder Samra, David Goltsman, Gideon Sandler, Matti L. Gild, Christian M. Girgis

**Affiliations:** 1 University of Sydney, Sydney, NSW Australia; 2 Westmead Hospital, Sydney, NSW Australia; 3 Royal North Shore Hospital, Sydney, NSW Australia

**Keywords:** Papillary thyroid cancer, Tall cell subtype, Early recurrence, Prognosis, Risk assessment, Retrospective studies

## Abstract

**Purpose:**

Tall cell papillary thyroid cancer (tcPTC) is traditionally considered to be an aggressive subtype of differentiated thyroid cancer, although its independent prognostic value is unclear. To investigate the independent prognostic value of tall cell morphology a tcPTC cohort was compared with a classical PTC (cPTC) cohort.

**Methods:**

A retrospective longitudinal study was performed using a cohort of tcPTC patients treated at Royal North Shore Hospital and Westmead Hospital in Sydney, Australia, and a cohort of cPTC patients treated at Westmead Hospital. Clinicopathological tumour characteristics and treatment pathways were analysed. Thyroglobulin and thyroglobulin antibody levels and further neck surgeries in the two years post thyroidectomy were used as a surrogate marker for early disease recurrence.

**Results:**

Presentation and treatment were analysed for 51 tcPTC patients and a comparator group of 365 cPTC patients. On univariate analysis, tcPTC was found to present at an older age (53.6 years v 46.4 years, *p* < 0.01), with greater rates of positive surgical margins (31.37% v 16.44%, *p* < 0.05), and greater rates of microscopic (47.06% v 22.74%, *p* < 0.001) and gross extrathyroidal extension (15.69% v 6.30%, *p* < 0.05). Longitudinal analysis was conducted for 236 patients (*n* = 24 for tcPTC, *n* = 212 for cPTC). Multivariate analysis found no difference in the odds of developing early recurrence between the tcPTC cohort and the cPTC cohort (OR = 0.65, *p* > 0.1).

**Conclusion:**

tcPTC is associated with more aggressive features compared with cPTC. Tall cell morphology was not found to be an independent predictor of early recurrence.

**Supplementary Information:**

The online version contains supplementary material available at 10.1007/s00405-025-09426-5.

## Introduction

**Fig. 1:**
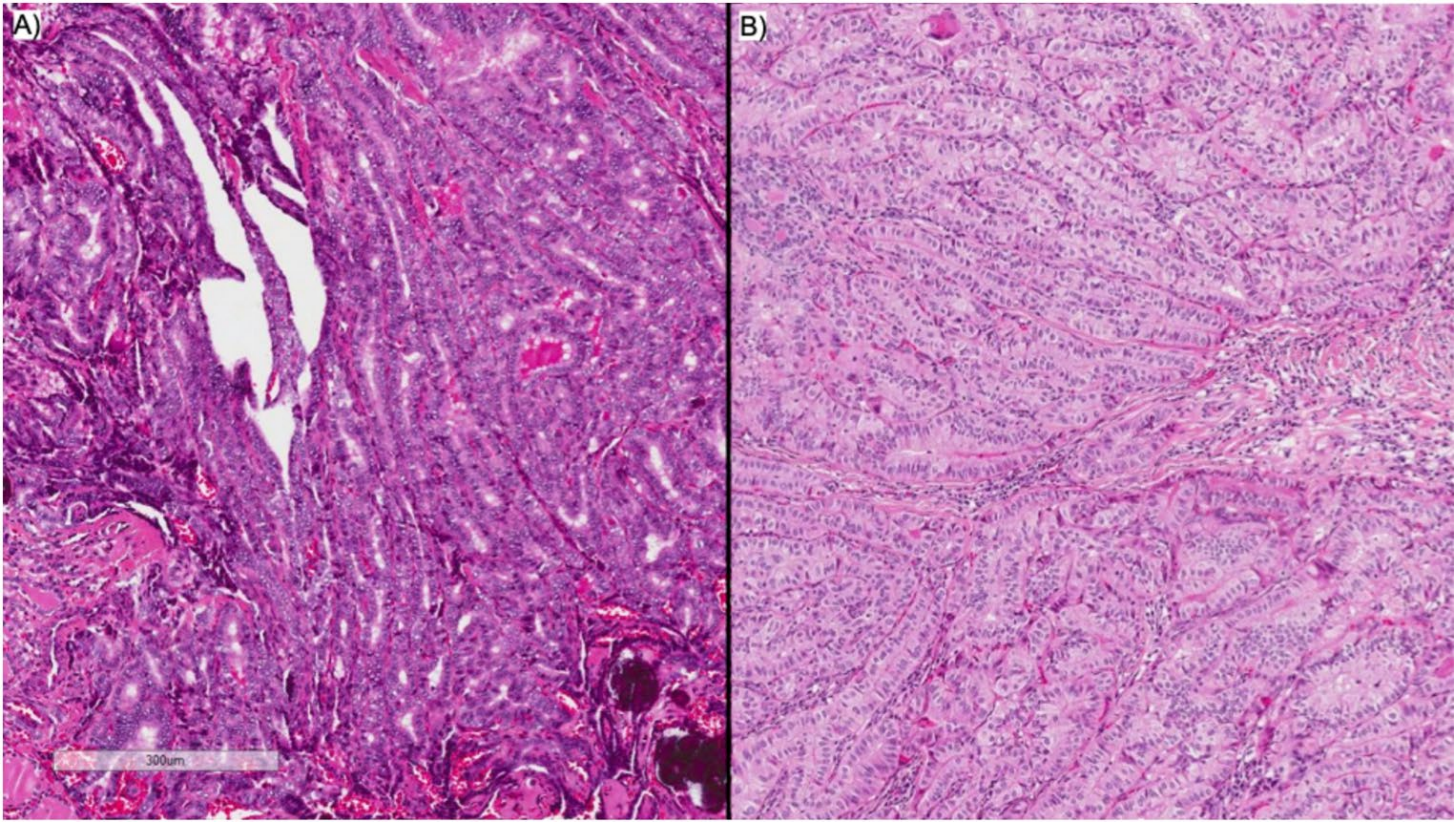
**A**) cPTC: This tumour shows classical architectural and cytomorphonuclear features of papillary carcinoma. There is nuclear overlapping and prominent nuclear clear with associated psammoma bodies. **B**) tcPTC: The tumour is composed of complex papillary and elongated trabecular formations that have a “tram track” appearance; the cells have a height-to-width ratio of at least 3:1 (H&E 40X)

Papillary thyroid cancer (PTC) is known as an indolent disease with excellent survival rates [1]. However, particular subtypes of PTC are considered to behave more aggressively. Of these aggressive subtypes, tall cell PTC (tcPTC) is the most common [2]. Recent WHO guidelines state that tall cells have a height at least three times the width of the cell, alongside an eosinophilic cytoplasm and tightly packed follicles and papillae [3]. A PTC must have at least 30% tall cells to be classified as tcPTC. A micrograph comparing classical PTC with tcPTC under haematoxylin and eosin staining is found in Fig. 1. ATA guidelines classify PTCs into low, intermediate and high risk of recurrence. The presence of tall cells, above the 30% threshold, renders these tumours as having an intermediate risk of recurrence and hence that they should receive more intensive treatment [4].

Intermediate risk of recurrence confers a recommendation for completion thyroidectomy and consideration of adjuvant radioactive iodine (RAI), whereas lobectomy alone may be adequate for low-risk PTC patients. RAI increases risk of secondary primary malignancies and salivary dysfunction, while completion thyroidectomies hold surgical risks such as recurrent laryngeal nerve injury, hypoparathyroidism, cervical haematoma and wound infection [5, 6]. As such, this upgrade to intermediate risk comes with significantly more morbidity.

Retrospective data suggests that tcPTC presents with more aggressive features than cPTC and is associated with a poorer outcome. tcPTC has been shown to present at an older age with larger tumour size, greater rates of extrathyroidal extension, higher rates of recurrence and poorer disease-specific survival [7–12]. However, there remains contention in the literature over whether this association is due to the tumours presenting at a later age and at a more advanced stage, or whether tcPTC can be considered to be an independently negative prognostic factor [7, 11–14].

Contributing to the lack of consensus on its prognostic value, histopathological criteria required for the diagnosis of tcPTC has changed several times over the past two decades. The 2004 WHO guidelines defined tcPTC as having > 50% tall cells with a height-to-width ratio (H: W) of > 3:1 [15]. In 2017, guidelines used a cut-off of 30% with a H: W of 2–3:1 [16]. The guidelines changed again in 2022, using a 30% cut-off with H: W > 3:1 [3]. These changes are outlined in Table 1. Further challenges to retrospective tcPTC studies are the significant interobserver variability in diagnosis of tcPTC [17] and continuing debate over the clinically significant tall cell proportion cut-off for diagnosis [18–21].

**Table 1:** Changes in histological definition of TcPTC

3^rd^ edition (2007)	4^th^ edition (2017)	5^th^ edition (2022)
> 50% tall cells	> 30% tall cells	> 30% tall cells
2-3x height: width	2-3x height: width	> 3x height: width

These changes have driven an increase in the incidence of tall cell diagnosis. The 2017 guidelines broadened the diagnostic net for tcPTC and were shown to increase incidence of tall cell diagnosis [22, 23]. Additionally, Ho et al. [8] found an increase in the incidence of aggressive subtypes of PTC over 2000 to 2016, at a greater rate than that of cPTC, which was driven by increasing awareness among pathologists. These changes suggest that tcPTC may have been underdiagnosed in retrospective studies that inform current guidelines.

Considering the heightened focus on intensive treatment for tcPTC patients, there is a requirement for up-to-date histopathological diagnostics to guide the development of optimal management guidelines in tcPTC. Here, we examined a tcPTC cohort and compared with a cPTC cohort to determine predictors for early recurrence. Early recurrence is defined based on ATA dynamic risk stratification guidelines [4], where it is defined as further surgery, as a proxy for incomplete structural response, or an incomplete biochemical response in the first two years post thyroidectomy. This approach allows for assessment of a contemporary cohort of patients to ascertain the role of tall cell histology in predicting outcome.

## Materials and methods

### Patient population

**Fig. 2:**
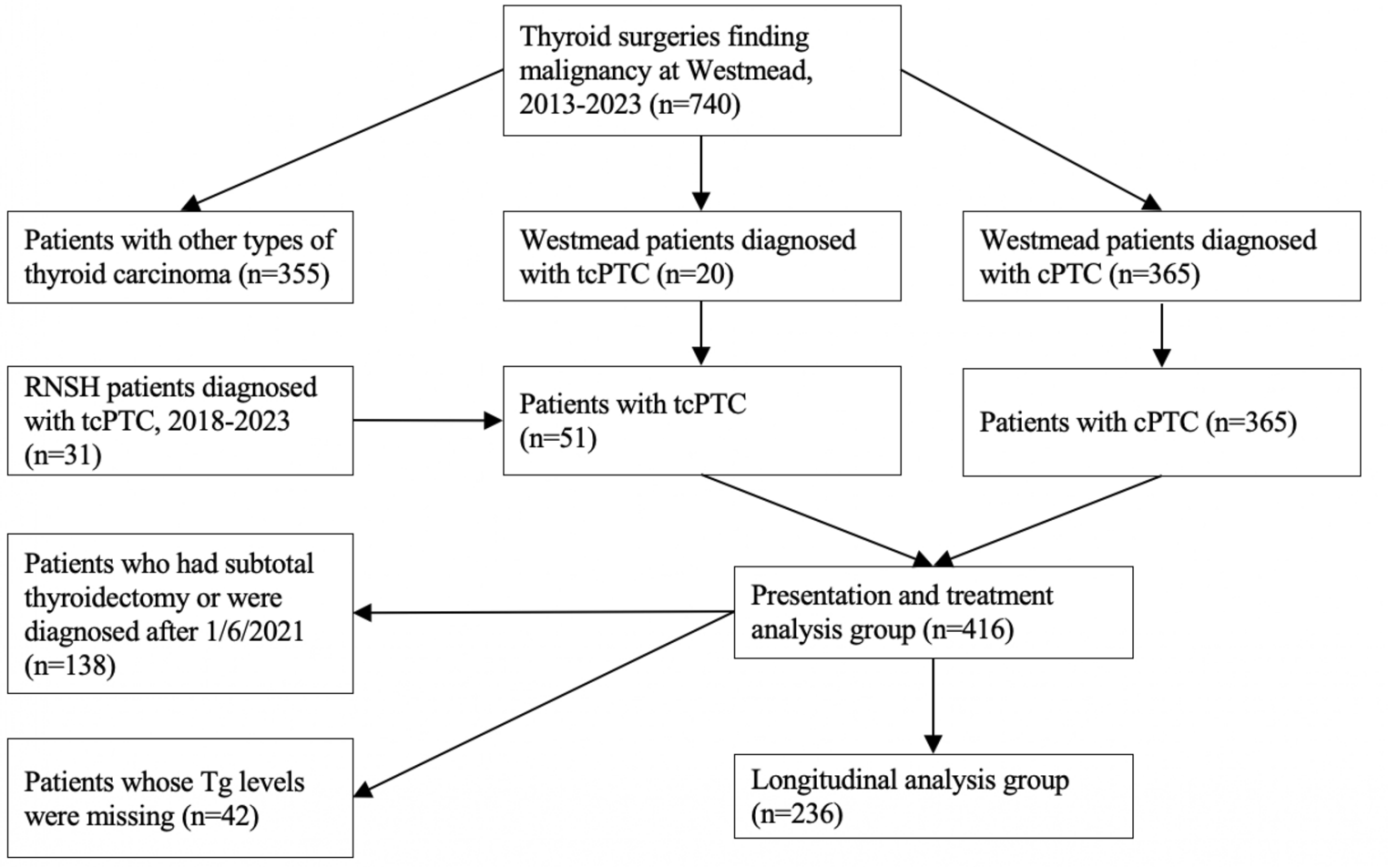
Patient recruitment flowchart

Using Western Sydney Local Health District surgical coding, 740 patients who had undergone thyroid-related surgeries for malignant neoplasms between January 1, 2013 and April 1, 2023 were identified. This list was cross referenced against an independent histopathology database at Westmead Hospital, coding for thyroid malignancy. From these two sets, the histology reports of 740 patients were scanned to determine thyroid cancer type and subtype. This process identified 365 patients with classical papillary thyroid cancer and 20 patients with tall cell subtype papillary thyroid cancer. Patients with other subtypes of papillary thyroid cancer, such as follicular subtype, were not included. A thorough analysis of electronic medical records was conducted for tcPTC and cPTC patients. All patients diagnosed with tcPTC at Westmead Hospital, and patients whose histology reports mentioned tall cell features, had their tumours reclassified according to the most recent 5th edition WHO guidelines [3]. In addition, a second site was used. Using the thyroid cancer database at Royal North Shore Hospital, another tertiary centre in Sydney, a diagnostic search for “tall cell” thyroid cancer was undertaken. This search yielded 31 additional cases of tcPTC diagnosed over 2018 to 2023. Presentation and treatment of the two tcPTC cohorts was compared to assess heterogeneity and suitability for grouping. This analysis is found in the supplementary information. There was found to be no significant difference between the two cohorts and they were therefore grouped together to increase the power of the study. Additionally, clinicians at both sites work closely together and therefore apply very similar management protocols. Longitudinal analysis was conducted for patients who underwent total or completion thyroidectomies. Patients were excluded if their surgery was prior to April 1, 2021, or if follow up blood biochemistry results could not be found. 236 patients fit inclusion criteria for longitudinal analysis. Figure 2 shows a patient recruitment flowchart. The study was approved by the Western Sydney Local Health District Scientific Advisory Committee (2304-06 QA) and by the Northern Sydney Local Health District Human Research Ethics Committee (2020/ETH02787).

### Clinicopathological variables

For each patient, data were extracted for age, gender, thyroid carcinoma type and subtype, date of admission, surgical procedure, tumour multifocality status, tumour size, involvement of surgical margins, lymphovascular invasion, perineural invasion, gross extrathyroidal extension (defined as invasion into perithyroidal skeletal muscle on histology), microscopic extrathyroidal extension, extra-nodal extension, T category and N category according to the American Joint Committee on Cancer 8th edition [24], RAI dose and date.

### Outcomes and assessments

Clinicopathological variables were used to contrast tumour characteristics between the tcPTC and cPTC groups. Data on surgical procedures and RAI treatment were used to establish differences in treatment protocols between the two groups. For subjects involved in longitudinal analysis, unstimulated thyroglobulin (Tg) and thyroglobulin antibody (TgAb) levels were recorded. Tg and TgAb levels were recorded for a maximum of three occasions over the initial follow up period of two years post-surgery. These data points were used to produce a biochemical response of excellent, incomplete, or indeterminate based on 2015 American Thyroid Association dynamic risk stratification guidelines [4]. Biochemical response was regarded as excellent when all thyroglobulin levels were below the lower limit of the assay (0.1ug/L for Abbott Architect, 0.9ug/L for Siemens Immulite). An indeterminate category was assigned when the most recent thyroglobulin antibody level was greater than 5IU/mL on the Abbott Architect method, or greater than 20IU/mL on the Siemens Immulite. When thyroglobulin antibody levels were regarded as not indeterminate and any thyroglobulin level was above the lower limit of the assay, biochemical response was regarded as incomplete. Patients with thyroglobulin levels below the lower limit of the assay but rising thyroglobulin antibodies were defined as excellent biochemical response, when TgAb levels were below the cutoff that would yield an indeterminate response. This decision was made in contrast with 2015 ATA guidelines due to the marginal increases in TgAb found in this small cohort of patients, such that the rise was regarded as clinically insignificant. A list of patients who had undergone further surgeries (not including completion thyroidectomies) in the two years post initial surgery was also recorded. Early recurrence was defined as an incomplete biochemical response or a further surgery in the two years post initial surgery, as a surrogate marker of structural recurrence.

### Statistical analysis

R version 4.3.1 and Excel version 16.74 were used for statistical analysis. Continuous variables are presented as means with sample standard deviation while categorical variables are presented as numbers with percentages. To contrast the tcPTC and cPTC cohorts, continuous and categorical variables are compared using independent two-sided t-tests and Pearson’s chi-squared tests, respectively. R library mass was used to perform logistic regression for multivariate analysis to produce odds ratios and 95% confidence intervals to determine risk factors associated with early recurrence. A p-value of < 0.05 was considered statistically significant.

## Results

51 tcPTC patients and 365 cPTC patients were included in this study, (416 patients). The Westmead Hospital cohort included 385 patients, 20 (5.48%) of which were classified as tcPTC. The Royal North Shore Hospital cohort included 31 tcPTC patients.

### Clinicopathological characteristics

Table 2 contrasts the tumour and clinical features of tcPTC patients and cPTC patients at presentation, illustrating that in our cohorts, tcPTC presented with more aggressive features. tcPTC presented with greater rates of microscopic extrathyroidal extension (47.06% v 22.74%, *p* < 0.001) and gross extrathyroidal extension (15.69% v 6.30%, *p* < 0.05) and were more likely to have positive surgical margins (31.37% v 16.44%, *p* < 0.05). tcPTC presented at a later age than cPTC (53.6 years v 46.4 years, *p* < 0.01). There was no statistically significant difference in lymph node positivity or lymph staging between the two cohorts, but there was a difference in T-stage (*p* < 0.05). Additionally, there was found to be no difference in multifocality between the two cohorts. A female preponderance was found in both cohorts (76.47% and 74.25%).

**Table 2:** Univariate analysis of TcPTC and cPTC cohorts

	Tall cell (*n* = 51)	Classical (*n* = 365)	*P*-value
Sex (female)	39 (76.47%)	271 (74.25%)	0.7
Age	53.61 (13.79)	46.43 (15.33)	0.002
Multifocality	25 (49.02%)	177 (48.49%)	0.9
Tumour size (mm)	20.68 (14.18)	16.96 (13.21)	0.06
Positive surgical margins	16 (31.37%)	60 (16.44%)	0.01
Microscopic extrathyroidal extension	24 (47.06%)	83 (22.74%)	0.0002
Gross extrathyroidal extension	8 (15.69%)	23 (6.3%)	0.02
Lymph node stage			0.7
NX	10 (19.61%)	135 (36.99)	
N0a	21 (41.18%)	100 (27.40%)	
N1a	13 (25.49%)	79 (21.64%)	
N1b	7 (13.73%)	51 (13.97%)	
T stage			0.02
T1a	10 (19.61%)	147 (40.27%)	
T1b	20 (39.22%)	116 (31.78%)	
T2	11 (21.57%)	61 (16.71%)	
T3a	3 (5.88%)	24 (6.58%)	
T3b	5 (9.80%)	14 (3.84%)	
T4a	2 (3.92%)	3 (0.82%)	

Used Pearson’s chi-squared test for categorical variables, independent samples t-test for continuous variables

Categorical variables are presented with percentage, continuous variables are presented with sample standard deviation

### Treatment protocols

Table 3 shows the treatments given to tall cell patients and to classical PTC patients following initial thyroid surgery. RAI was more frequently administered to tall cell patients (66.7% v 41.9%, *p* < 0.05). RAI doses were higher for tall cell patients (3.64Gbq v 3.23Gbq, *p* = 0.1), though not to the level of statistical significance. Completion thyroidectomies were more often performed for tall cell patients (21.6% v 4.7%).

**Table 3:** Treatment protocols

	Tall cell (*n* = 51)	Classical (*n* = 365)	*P*-value
RAI	34 (66.7%)	153 (41.9%)	0.0009
Average dose (Giga-becquerels)	3.64 (1.67)	3.23 (1.22)	0.1
Surgery performed			0.00006
Completion thyroidectomy	11 (21.6%)	17 (4.7%)	
Total thyroidectomy	36 (70.6%)	281 (77.0%)	
Hemithyroidectomy	4 (7.8%)	64 (17.5%)	
Isthmusectomy	0	3 (0.8%)	

### Longitudinal analysis

Longitudinal analysis was conducted for 236 patients, including 24 tall cell patients and 212 classical PTC patients. Table 4 shows that of the tall cell cohort, 10 (42%) had a biochemically excellent response, 8 (33.33%) had a biochemically incomplete response and 6 (25%) had an indeterminate response. Of the classical cohort, 96 (45%) had an excellent response, 80 (38%) had an incomplete response and 36 (17%) had an indeterminate response. There was no statistically significant difference between these two groups (*P* = 0.62). In the two years following initial surgery, 3 (13%) further surgeries were performed for tcPTC patients and 10 (5%) further surgeries were performed for cPTC patients, excluding completion thyroidectomies. 12 of these surgeries were for excision of cervical lymph nodes and 1 surgery was for excision of paratracheal lymph nodes.

**Table 4:** Longitudinal analysis

	Tall cell (*n* = 24)	Classical (*n* = 212)	*P*-value
Biochemical response			0.6
Excellent	10 (41.67%)	96 (45.28%)	
Incomplete	8 (33.33%)	80 (37.74%)	
Indeterminate	6 (25%)	36 (16.98%)	
Further surgeries	3 (12.5%)	10 (4.72%)	0.11
Early recurrence	11 (45.83%)	90 (42.45%)	0.8

Table 5 shows the odds ratios for developing early recurrence. Variables included in the logistic regression model were those listed in Table 5. Statistically significant odds ratios were found between patients with and without positive surgical margins (OR = 2.84, *p* < 0.005) and between patients with and without lymphovascular invasion (OR = 2.73, *p* < 0.005). There was no statistically significant difference in the odds ratio of developing early recurrence between patients with tcPTC and patients with cPTC (OR = 0.65, *p* = 0.4).

**Table 5:** Odds ratios for early recurrence

	Odds ratio (95% CI)	*P*-value
Age > 45	0.84 (0.45–1.54)	0.6
Female	0.79 (0.40–1.56)	0.5
Tall cell	0.65 (0.23–1.73)	0.4
Tumour size	1.00 (0.98–1.02)	0.9
Positive surgical margins	2.84 (1.40–5.86)	0.004
Multifocality	1.04 (0.57–1.92)	0.9
Lymphovascular invasion	2.73 (1.45–5.18)	0.002
Gross extrathyroidal extension	1.30 (0.50–3.43)	0.6
Lymph-node positive	1.81 (0.93–3.52)	0.08
RAI dose	1.02 (0.85–1.22)	0.8

## Discussion

This retrospective study adds to the body of evidence that tcPTC presents with more aggressive characteristics than cPTC. Here, we describe the outcomes of a cPTC and tcPTC cohort in the last 10 years at Westmead Hospital, and the outcomes of a tcPTC cohort in the last 5 years at Royal North Shore Hospital. In the Westmead cohort, the proportion of tcPTC of all PTC was 3.9%. Compared to the cPTC cohort, statistically significant differences in age, involvement of margins, microscopic and gross extrathyroidal extension and T-stage were found between the tcPTC cohort and the cPTC cohort. This replicates findings made across single and multi-centre studies [11, 12, 25], as well as large database studies of tcPTC [7–10].

This main aim of this study was to determine the independent prognostic value of tall cell morphology within these retrospective cohorts. National database studies on tcPTC, such as those by Kazaure et al. [9] and Ho et al. [8], have used overall survival to assess patient outcomes however in practice we look at recurrence risks and morbidity, rather than looking at predictors of the very low rates of mortality. In addition, the relative rarity of tcPTC together with its slow disease course requires lengthy periods of assessment and follow up, over which time awareness and definition of the disease may have changed markedly [3, 15–17]. This may explain the lack of consensus as to its status as an independently negative prognostic factor. Here, a shorter, two year follow up period allowed for the assessment of a contemporary cohort and practically can assist in providing guidance for patients for their risk of early recurrence.

The prognostic role of tall cells was assessed using logistic regression to determine factors associated with early recurrence, using a physiological selection of variables to control for the more aggressive features associated with tcPTC and RAI dose. Early recurrence was defined as an incomplete biochemical response or further surgery in the two years post initial surgery, in line with dynamic risk stratification put forward in the 2015 ATA guidelines [4].

In our study, we found no difference in the odds of developing early recurrence between patients with tcPTC and cPTC. This suggests that independent of baseline characteristics and RAI dose, tall cell subtype histology itself is not associated with poorer outcomes. Thereby, upgrading patients from low risk of structural recurrence to intermediate risk based on tall cell histology may not be warranted, given that patients who may otherwise be low risk will then be exposed to the morbidity associated with more aggressive treatment. Additionally, this is reassuring for patients, who are unlikely to have an early recurrence purely due to having tcPTC.

Single centre retrospective studies by Song et al. [12] and Regalbuto et al. [26] also found tall cell morphology not to be an independently negative prognostic factor. Recent studies suggesting that tcPTC could be treated more conservatively include a 2021 paper by Limberg et al. [2], which suggested that in the absence of aggressive features, tcPTC has comparable outcomes to cPTC, and a 2023 paper by Woods et al. [27], which found that node negative, T1/T2 tcPTC can be adequately managed with lobectomy.

A major limitation of this study is the size of the cohort included. We note that the multivariate analysis failed to show a statistically significant difference in odds for several variables regarded as being predictive of a poorer disease course, such as advanced age and gross extrathyroidal extension. The latter may be due to the study using a histological rather than surgical definition for gross extrathyroidal extension. Our finding that tall cell morphology was not predictive of early recurrence would have been more strongly supported if other variables traditionally associated with further disease had been found to be predictive of early recurrence in our model. This unexpected finding could demonstrate that the study may be underpowered, and that a greater number of participants would be required to confirm these additional risk factors. Furthermore, the follow up period of two years may be not long enough to allow for later recurrence that can occur in PTC.

Using further surgery as a proxy for structural response could fail to capture patients with distant metastatic disease who do not go on to have further surgery. However, we would expect that thyroglobulin levels would detect recurrence in these patients. Including structural response, based on imaging findings, would allow for clearer differentiation between those with incomplete structural response and a purely biochemical incomplete response. We note that analysis of patients who developed distant metastatic disease would be of value, given the benign prognosis for PTC patients even after further RAI or additional surgery such as cervical lymph node excision. This would however require a much larger sample size, given the rarity of metastatic disease in PTC. We call for further studies to address this.

Centralised histological overview of all cases using 5th edition WHO guidelines was not performed across both centres, reflecting the ‘real world’ nature of this study. Significant interobserver variability in tall cell diagnosis is well known [17], as well as in other aspects of histological classification [28, 29]. Although all Westmead cases featuring tall cells were reviewed, it is possible that a small number of cPTC cases in the dataset would now be classified as tcPTC. The impact of the 2022 changes in definition has not yet been assessed in the literature and thus the consequences of a reclassification according to 5th edition guidelines cannot be predicted. The difference in tcPTC diagnosis across time, observers, and institutions is a problem common to all retrospective tcPTC studies, especially those using national databases. Larger prospective studies are needed in this field to further confirm our findings and to inform risk stratification guidelines.

This study was unique in using a shorter follow up period alongside a definition for early recurrence based on dynamic risk stratification guidelines. This may assist clinicians in reassuring patients with tall cell histology that the inherent presence of these cells is not necessarily predictive of worse outcome. In this retrospective, dual centre cohort study, we showed tcPTC presents with more aggressive features than cPTC, in keeping with prior literature. Additionally, multivariate analysis found positive surgical margins and lymphovascular invasion to be predictive of early recurrence but did not find tall cell histology to be predictive of early recurrence. Clinically, we suggest that tall cell features alone may not warrant more intensive treatment, such as completion thyroidectomy and RAI treatment. Aggressive treatment may not be necessary for all tall cell patients and clinicians should personalise management accordingly.

## Electronic supplementary material

Below is the link to the electronic supplementary material.

Supplementary Material 1

## References

[1] Davies L, Welch HG (2014) Current thyroid cancer trends in the united States. JAMA Otolaryngol Head Neck Surg 140(4):317–322. 10.1001/jamaoto.2014.1 24557566 10.1001/jamaoto.2014.1

[2] Limberg J, Ullmann TM, Stefanova D, Buicko JL, Finnerty BM, Zarnegar R et al (2021) Does aggressive variant histology without invasive features predict overall survival in papillary thyroid cancer?? A National cancer? database analysis. Ann Surg 274(3):e276–e81. 10.1097/SLA.0000000000003632 31599802 10.1097/SLA.0000000000003632

[3] Baloch ZW, Asa SL, Barletta JA, Ghossein RA, Juhlin CC, Jung CK et al (2022) Overview of the 2022 WHO classification of thyroid neoplasms. Endocr Pathol 33(1):27–63. 10.1007/s12022-022-09707-3 35288841 10.1007/s12022-022-09707-3

[4] Haugen BR, Alexander EK, Bible KC, Doherty GM, Mandel SJ, Nikiforov YE et al (2016) 2015 American thyroid association management guidelines for adult patients with thyroid nodules and differentiated thyroid cancer: the American thyroid association guidelines task force on thyroid nodules and differentiated thyroid Cancer. Thyroid 26(1):1–133. 10.1089/thy.2015.0020 26462967 10.1089/thy.2015.0020 PMC4739132

[5] Iyer NG, Morris LG, Tuttle RM, Shaha AR, Ganly I (2011) Rising incidence of second cancers in patients with low-risk (T1N0) thyroid cancer who receive radioactive iodine therapy. Cancer 117(19):4439–4446. 10.1002/cncr.26070 21432843 10.1002/cncr.26070 PMC3155861

[6] Mandel SJ, Mandel L (2003) Radioactive iodine and the salivary glands. Thyroid 13(3):265–271. 10.1089/105072503321582060 12729475 10.1089/105072503321582060

[7] Morris LG, Shaha AR, Tuttle RM, Sikora AG, Ganly I (2010) Tall-cell variant of papillary thyroid carcinoma: a matched-pair analysis of survival. Thyroid 20(2):153–158. 10.1089/thy.2009.0352 20151822 10.1089/thy.2009.0352 PMC3714453

[8] Ho AS, Luu M, Barrios L, Chen I, Melany M, Ali N et al (2020) Incidence and mortality risk spectrum across aggressive variants of papillary thyroid carcinoma. JAMA Oncol 6(5):706–713. 10.1001/jamaoncol.2019.6851 32134428 10.1001/jamaoncol.2019.6851 PMC7059113

[9] Kazaure HS, Roman SA, Sosa JA (2012) Aggressive variants of papillary thyroid cancer: incidence, characteristics and predictors of survival among 43,738 patients. Ann Surg Oncol 19(6):1874–1880. 10.1245/s10434-011-2129-x 22065195 10.1245/s10434-011-2129-x

[10] Axelsson TA, Hrafnkelsson J, Olafsdottir EJ, Jonasson JG (2015) Tall cell variant of papillary thyroid carcinoma: a population-based study in Iceland. Thyroid 25(2):216–220. 10.1089/thy.2014.0075 25322334 10.1089/thy.2014.0075

[11] Michels JJ, Jacques M, Henry-Amar M, Bardet S (2007) Prevalence and prognostic significance of tall cell variant of papillary thyroid carcinoma. Hum Pathol 38(2):212–219. 10.1016/j.humpath.2006.08.001 17097131 10.1016/j.humpath.2006.08.001

[12] Song E, Jeon MJ, Oh HS, Han M, Lee YM, Kim TY et al (2018) Do aggressive variants of papillary thyroid carcinoma have worse clinical outcome than classic papillary thyroid carcinoma? Eur J Endocrinol 179(3):135–142. 10.1530/EJE-17-0991 29875289 10.1530/EJE-17-0991

[13] Silver CE, Owen RP, Rodrigo JP, Rinaldo A, Devaney KO, Ferlito A (2011) Aggressive variants of papillary thyroid carcinoma. Head Neck 33(7):1052–1059. 10.1002/hed.21494 20824810 10.1002/hed.21494

[14] Gunalp B, Okuyucu K, Ince S, Ayan A, Alagoz E (2017) Impact of tall cell variant histology on predicting relapse and changing the management of papillary thyroid carcinoma patients. Hell J Nucl Med 20(2):122–127. 10.1967/s002449910552 28697188 10.1967/s002449910552

[15] Lloyd R, De Lellis R, Heitz P, Eng C (2004) World health organization classification of tumours: pathology and genetics of tumours of the endocrine organs. International Agency for Research on Cancer (IARC), Lyon

[16] Lloyd RV, Osamura RY, Klöppel G, Rosai J (2017) WHO classification of tumours of endocrine organs. 4th edition ed

[17] Hernandez-Prera JC, Machado RA, Asa SL, Baloch Z, Faquin WC, Ghossein R et al (2017) Pathologic reporting of Tall-Cell variant of papillary thyroid cancer: have we reached a consensus?? Thyroid 27(12):1498–1504. 10.1089/thy.2017.0280 29020884 10.1089/thy.2017.0280

[18] Ganly I, Ibrahimpasic T, Rivera M, Nixon I, Palmer F, Patel SG et al (2014) Prognostic implications of papillary thyroid carcinoma with tall-cell features. Thyroid 24(4):662–670. 10.1089/thy.2013.0503 24262069 10.1089/thy.2013.0503

[19] Bongers PJ, Kluijfhout WP, Verzijl R, Lustgarten M, Vermeer M, Goldstein DP et al (2019) Papillary thyroid cancers with focal tall cell change are as aggressive as tall cell variants and should not be considered as Low-Risk disease. Ann Surg Oncol 26(8):2533–2539. 10.1245/s10434-019-07444-2 31115855 10.1245/s10434-019-07444-2

[20] Stenman S, Siironen P, Mustonen H, Lundin J, Haglund C, Arola J (2018) The prognostic significance of tall cells in papillary thyroid carcinoma: A case-control study. Tumour Biol 40(7):1010428318787720. 10.1177/1010428318787720 30010512 10.1177/1010428318787720

[21] Bikas A, Wong K, Pappa T, Ahmadi S, Wakefield CB, Marqusee E et al (2023) Papillary thyroid carcinomas with tall cell features: an intermediate entity between classic and tall cell subtypes. Thyroid. 10.1089/thy.2022.0534 36960703 10.1089/thy.2022.0534

[22] Poma AM, Viola D, Macerola E, Proietti A, Molinaro E, De Vietro D et al (2021) Tall cell percentage alone in PTC without aggressive features should not guide patients’ clinical management. J Clin Endocrinol Metab 106(10):e4109–e17. 10.1210/clinem/dgab388 34061965 10.1210/clinem/dgab388

[23] Wong KS, Higgins SE, Marqusee E, Nehs MA, Angell T, Barletta JA (2019) Tall cell variant of papillary thyroid carcinoma: impact of change in WHO definition and molecular analysis. Endocr Pathol 30(1):43–48. 10.1007/s12022-018-9561-4 30565013 10.1007/s12022-018-9561-4

[24] Amin MB, Edge SB, Greene FL, Byrd DR, Brookland RK, Washington MK et al (2018) AJCC Cancer Staging Manual: Springer International Publishing

[25] Longheu A, Canu GL, Cappellacci F, Erdas E, Medas F, Calo PG (2020) Tall cell variant versus conventional papillary thyroid carcinoma: A retrospective analysis in 351 consecutive patients. J Clin Med 10(1). 10.3390/jcm10010070 10.3390/jcm10010070 PMC7794904 33379135

[26] Regalbuto C, Malandrino P, Frasca F, Pellegriti G, Le Moli R, Vigneri R et al (2013) The tall cell variant of papillary thyroid carcinoma: clinical and pathological features and outcomes. J Endocrinol Invest 36(4):249–254. 10.3275/8515 22776915 10.3275/8515

[27] Woods RSR, Fitzgerald CWR, Valero C, Lopez J, Morris LGT, Cohen MA et al (2023) Surgical management of T1/T2 node-negative papillary thyroid cancer with tall cell histology: is lobectomy enough? Surgery. 173(1):246–251. 10.1016/j.surg.2022.05.045 10.1016/j.surg.2022.05.045 36257862

[28] Su HK, Wenig BM, Haser GC, Rowe ME, Asa SL, Baloch Z et al (2016) Inter-Observer variation in the pathologic identification of minimal extrathyroidal extension in papillary thyroid carcinoma. Thyroid 26(4):512–517. 10.1089/thy.2015.0508 26953223 10.1089/thy.2015.0508 PMC5583558

[29] Du E, Wenig BM, Su HK, Rowe ME, Haser GC, Asa SL et al (2016) Inter-Observer variation in the pathologic identification of extranodal extension in nodal metastasis from papillary thyroid carcinoma. Thyroid 26(6):816–819. 10.1089/thy.2015.0551 27089928 10.1089/thy.2015.0551

