# Supplementary material for: Biochemical and structural response in patients with tall cell papillary thyroid cancer: a dual centre retrospective study: Early Recurrence in Tall Cell PTC
Source: Eur Arch Otorhinolaryngol. 2025 May 5;282(8):4265–72. doi: 10.1007/s00405-025-09426-5 (PMC12399698; doi:10.1007/s00405-025-09426-5)
Supplement: Supplementary file 1 — Supplementary Material 1 [file 405_2025_9426_MOESM1_ESM.docx]

FOR SUPPLEMENTARY MATERIAL

Table 6 - Univariate analysis of tcPTC cohorts

|  | RNSH (n=31) | Westmead (n=20) | P-value |
| --- | --- | --- | --- |
| Sex (Female) | 23 (74.19%) | 16 (80.00%) | 0.6 |
| Age | 55.03 (13.76) | 51.40 (13.90) | 0.4 |
| Multifocality | 14 (45.16%) | 11 (55.00%) | 0.5 |
| Tumour size (mm) | 19.99 (14.07) | 21.75 (14.64) | 0.7 |
| Margins | 13 (41.94%) | 3 (15.00%) | 0.04 |
| Micro ETE | 14 (45.16%) | 10 (50.00%) | 0.7 |
| ETE gross | 4 (12.90%) | 4 (20.00%) | 0.5 |
| Lymph node stage | | | 0.3 |
| NX | 5 (16.13%) | 5 (25.00%) |  |
| N0a | 16 (51.61%) | 5 (25.00%) |  |
| N1a | 7 (22.58%) | 6 (30.00%) |  |
| N1b | 3 (9.68%) | 4 (20.00%) |  |
| T stage | | | 0.6 |
| T1a | 7 (22.58%) | 3 (15.00%) |  |
| T1b | 12 (38.71%) | 8 (40.00%) |  |
| T2 | 7 (22.58%) | 4 (20.00%) |  |
| T3a | 1 (3.23%) | 2 (10.00%) |  |
| T3b | 2 (6.45%) | 3 (15.00%) |  |
| T4a | 2 (6.45%) | 0 (0.00%) |  |
| RAI | 20 (64.52%) | 14 (70.00%) | 0.7 |
| RAI dose | 3.85 (1.80) | 3.22 (1.37) | 0.3 |
| Procedure | | | 0.1 |
| Completion thyroidectomy | 8 (25.81%) | 3 (15.00%) |  |
| Total thyroidectomy | 19 (61.29%) | 17 (85.00%) |  |
| Hemithyroidectomy | 4 (12.90%) | 0 (0.00%) |  |
| Used Pearson's chi-squared test for categorical variables, independent samples t-test for continuous variables | | | |
| Categorical variables are presented with percentage, continuous variables are presented with sample standard deviation | | | |
